# Association between serum albumin and preoperative Deep Vein Thrombosis in patients aged 80 and over with intertrochanteric fracture: A retrospective observational study

**DOI:** 10.1371/journal.pone.0345895

**Published:** 2026-08-14

**Authors:** Caizhen Chen, Xiuguo Zhang

**Affiliations:** Department of Nursing, The Third Hospital of Hebei Medical University, Shijiazhuang, Hebei, China; Siloam Hospitals Lippo Village, INDONESIA

## Abstract

**Objective:**

The association between serum albumin level and preoperative deep vein thrombosis (DVT) in patients aged ≥ 80 years with intertrochanteric fractures remains unclear. This study aimed to examine the association between serum albumin level and preoperative DVT and to explore whether the relationship between serum albumin and DVT exhibits a nonlinear pattern.

**Methods:**

This retrospective observational study included 1484 consecutive patients aged ≥ 80 years with intertrochanteric fractures who were admitted to our hospital between January 2016 and July 2024. Vital signs, laboratory data, and comorbidities were collected for all participants to examine the association between serum albumin and preoperative DVT. To assess the independent association between serum albumin and DVT, we employed multivariable logistic regression analyses and smooth curve fitting. Subgroup analyses and interaction tests were conducted, followed by sensitivity analyses using propensity score matching.

**Results:**

A total of 1484 patients aged ≥ 80 years with intertrochanteric fractures were included in the study. After adjusting for potential confounders, each 1 g/L higher serum albumin level was associated with 5% lower odds of preoperative DVT (odds ratio [OR], 0.95; 95% confidence interval [CI], 0.92–0.97, *P* < 0.01). A linear relationship was observed between serum albumin and preoperative DVT, and participants in the highest quartile of serum albumin had significantly lower preoperative DVT than those in the lowest quartile. Similar patterns were observed in subgroup analyses, with significant interaction effects noted for coronary heart disease, and sensitivity analyses showed that the results were robust.

**Conclusions:**

Reduced serum albumin concentrations demonstrate a significant association with elevated preoperative DVT in patients aged ≥80 years with intertrochanteric fractures, and further studies are required to confirm this relationship.

## Introduction

Hip fractures are a major public health problem associated with increased disability, healthcare costs, and mortality [1,2]. The number of patients with hip fractures is projected to increase by 6.26 million by 2050, and more than half of these patients will have intertrochanteric fractures [3–5]. Intertrochanteric fractures are common osteoporosis-related fractures among the older population, accounting for 10%–20% of all fractures [6] and constituting 50%–65% of hip fractures [7]. With a 30-day mortality rate ranging from 1.0% to 6.5% and a 1-year mortality rate increasing considerably to 37.3%, intertrochanteric fractures represent the primary cause of death in the older population [8]. A prospective cohort study has reported that nonagenarian patients with intertrochanteric fractures exhibited mortality rates of 7.6%, 13.9%, and 28.5% at 6-month, 1-year, and 2-year follow-ups, respectively [9]. As the population ages, intertrochanteric fractures carry significant risks of serious complications, such as deep vein thrombosis (DVT), nonunion of the fracture, and femoral head necrosis, which can be fatal in extreme circumstances [10].

Preoperative DVT is one of the most complications after intertrochanteric fracture that affects 8.0%–34.9% of the older patients [11–13] due to a hypercoagulable state and immobilization. DVT, a thromboembolic event, can progress proximally into pulmonary embolism (PE) and can be fatal [14].

The pathophysiology of thrombus formation involves three key factors: stasis or blood flow changes, blood hypercoagulation, and vascular wall injury. Alterations in the coagulation system that induce a hypercoagulable state are risk factors for thrombosis development, and elevated concentrations of coagulation factors II, VIII, IX, XII, and fibrinogen are independent risk factors for VTE [15–17]. Additionally, low albumin levels play a significant role in DVT development [18,19]. Albumin suppresses both fibrin polymerization and platelet aggregation and exhibits heparin-like characteristics [20]. Furthermore, reduced serum albumin concentrations may result in diminished plasma colloidal osmotic pressure, elevated blood viscosity, and eventually, thrombus formation [21]. Previous research has highlighted the significance of serum albumin levels for predicting the prognosis and complications in orthopedic patients before surgery [19]; among older patients with hip fractures, lower serum albumin levels increase susceptibility to perioperative complications and are independently linked to preoperative DVT development [22–24].

However, the specific relationship between albumin and preoperative DVT, particularly among octogenarian patients, as well as the nonlinear relationship between them remain unclear. Therefore, this study aimed to elucidate the association between albumin and preoperative DVT in patients aged ≥80 years with intertrochanteric fractures.

## Materials and methods

### Ethics statement

This study was approved by the Institutional Review Board of the ethics committee of the Third Hospital of Hebei Medical University (approval no. W2023-064-2). Patient informed consent was waived owing to the retrospective design of this study, and data were accessed for research purpose on 12/01/2026. All data were anonymized prior to analysis, which entailed minimal risk to the participants. Comprehensive measures were implemented to ensure patient confidentiality and the protection of personal data.

### Study population

This retrospective study included consecutive patients aged ≥80 years who were diagnosed with intertrochanteric femoral fractures and admitted to the Department of Geriatric Orthopedics, The Third Hospital of Hebei Medical University between January 2016 and July 2024. Serum albumin was measured on admission within 24 h, and Doppler ultrasonography (DUS) was performed for DVT screening prior to surgery. The inclusion criteria were as follows: (1) intertrochanteric femoral fractures; (2) age ≥ 80 years; (3) discontinuity or significant displacement of the cortical bone between the intertrochanteric region on X-ray or computed tomography scans, as well as significant clinical symptoms in the hip following trauma; (4) independent pre-fracture mobility, undergoing DUS for detection of preoperative DVT, and receiving definite surgical care. The exclusion criteria were as follows: (1) multiple fractures or pathological/metastatic fractures; (2) immunological system (e.g., systemic lupus erythematosus, antiphospholipid syndrome) or hematological abnormalities present (e.g., inherited thrombophilia, myeloproliferative neoplasms); (3) fractures caused by high-energy injury; (4) open fractures; (5) liver failure or nephrotic syndrome; (6) diagnosis of venous thromboembolism (VTE) before injury; and (7) incomplete data.

### Exposure

Hypoalbuminemia, defined as a serum albumin level < 35 g/L, is a common clinical condition typically characterized by serum albumin concentrations below the normal range [25]. Blood samples from patients with hip fractures were collected within 24 h of admission to determine the presence of baseline hypoalbuminemia.

### Diagnosis and management of DVT

Lower extremity ultrasonography was performed by experienced radiologists who were blinded to laboratory findings. DVT was diagnosed by duplex ultrasonography. Diagnosis was established based on the Rabinov group criteria [26], requiring identification of venous lumen obstruction or filling defects. Key ultrasonographic features encompassed venous noncompressibility, luminal obstruction or filling defects, absence of respiratory variation in above-knee venous segments, and impaired flow augmentation in calf and foot veins during compression maneuvers [27]. According to our institutional policy, for major orthopedic trauma (such as hip fractures) or before major orthopedic surgery, suspected or high-risk DVT must be detected through DUS or venography of the lower extremities, to reduce the occurrence of severe adverse events. Upon each patient’s admission, elevation of the injured limb, quadriceps strengthening exercises, and ankle pump exercises are to be performed. Prophylactic low-molecular-weight heparin (LMWH) was initiated within 24h of admission once the bleeding risk was adequately controlled. Intermittent pneumonic compression (IPC) was routinely used as indicated.

### DVT classification

DVT was classified into two types according to the anatomical location of the thrombus: proximal and distal. Proximal DVT was defined as thrombosis occurring in the popliteal vein or at the proximal end, such as thrombosis in the iliac vein, femoral vein, deep femoral vein, common femoral vein or popliteal vein. Distal DVT was defined as thrombosis occurring at the distal end of the knee joint, including thrombosis of the tibia, the fibular vein or intermuscular vein. The combination of distal and proximal thrombi is classified as proximal thrombus.

### Covariable

All data were extracted from the patients’ hospitalization medical records and related to demographics (age, sex, marital status, and history of VTE), lifestyle (smoking and alcohol consumption), comorbidities (hypertension, diabetes, coronary artery disease, and renal insufficiency), injury-related data (time of injury to the hospital), and laboratory results [platelet count (PLT), fibrinogen (FIB), thrombin time (TT), prothrombin time (PT), D-dimer, total cholesterol (TC) and triglycerides (TG)].

### Statistical analysis

Continuous variables are summarized as means ± standard deviations (SD) for normal distributions or medians and interquartile ranges for skewed distributions, and categorical variables are expressed as frequencies (percentages). We used the chi-square test, one-way analysis of variance, and Kruskal–Wallis test to compare categorical, normally distributed, and nonnormally distributed continuous variables.

To assess the independent relationship between albumin level and preoperative DVT, we used multivariate logistic regression models to determine the odds ratios (ORs) and 95% confidence intervals (CIs). We controlled for significant covariates selected based on expert judgment and relevant literature [13,28,29]. Four models were used to explore the relationships. Model 1 was unadjusted, whereas Model 2 was adjusted for age, sex, and marital status. Model 3 was further adjusted for hypertension, diabetes, coronary artery disease, renal insufficiency, history of VTE, smoking, alcohol consumption, and time from injury to hospital admission. Model 4 included additional adjustments for PLT, FIB, TT, APTT, PT, D-dimer, TC, and TG; and the E-value was used to evaluate the influence of unmeasured confounding factors on the research results [30].

Additionally, restricted cubic spline (RCS) regression was employed with four knots at the 5th, 35th, 65th, and 95th percentiles of serum albumin to evaluate linearity and examine the dose–response curve between albumin and preoperative DVT after adjusting for variables in Model 4. Subgroup analyses were performed, and interactions between subgroups were assessed using the likelihood ratio test. All statistical analyses were performed using R 4.2.2 (http://www.Rproject.org; The R Foundation, Vienna, Austria) and Free Statistics software (version 2.3; Beijing Free Clinical Medical Technology Co., Ltd, Beijing, China). A two-sided test, *P* < 0.05, was considered statistically significant.

## Results

### Participants’ characteristics

**Fig 1:**
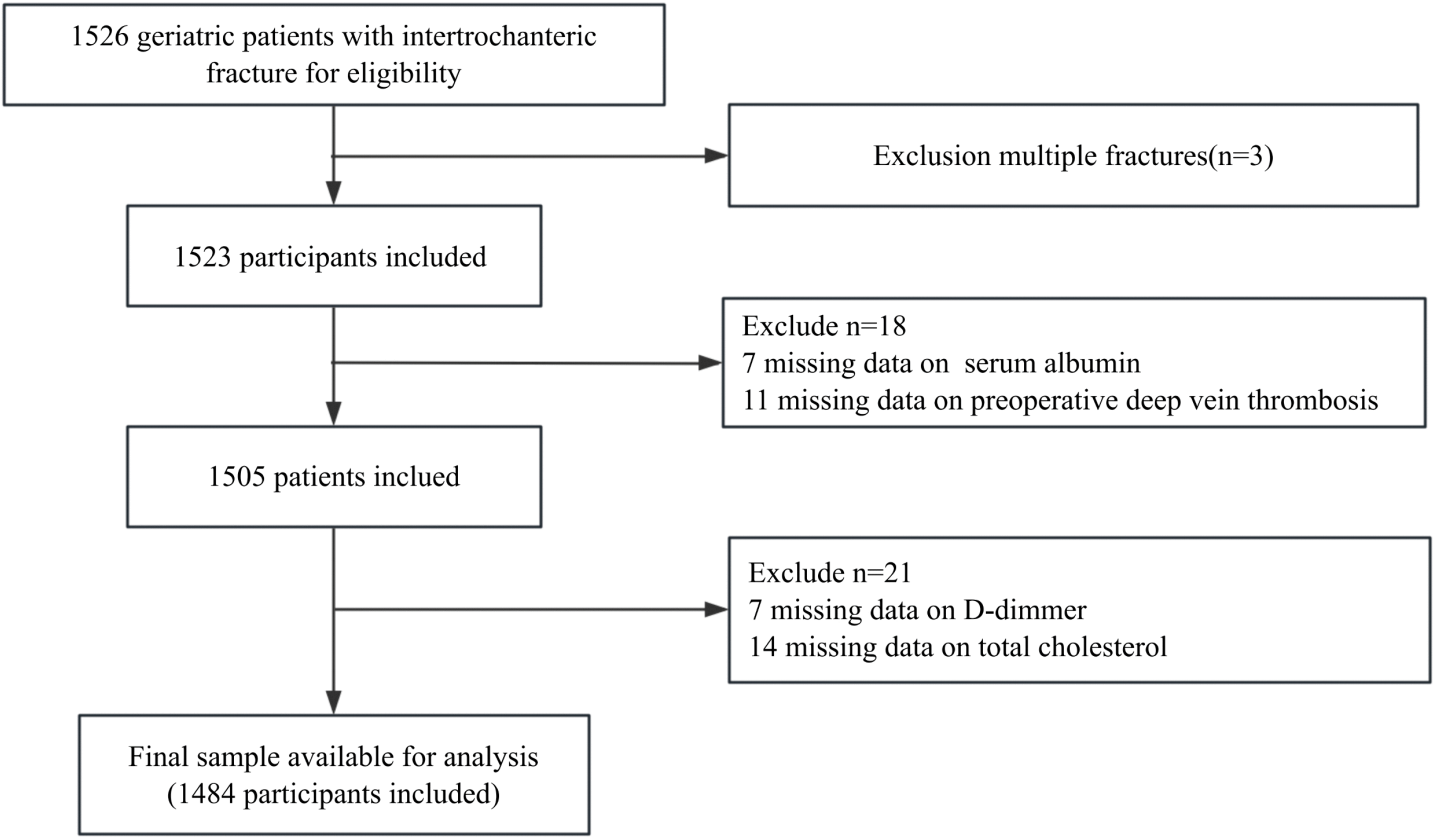
Flow chart of the study. Abbreviations: FIB, Fibrinogen; TT, Thrombin Time; APTT activated partial thromboplastin time; PT, Prothrombin Time; ALB, Albumin; TG, Triglyceride; TC, Total Cholesterol.

A total of 1484 eligible patients (366 men and 1118 women) were enrolled based on the study criteria (Fig 1). The average age was 85.7 ± 4.2 years (range, 80–106 years). The preoperative serum albumin was 35.39 ± 4.16 g/L. A total of 655 patients were diagnosed with preoperative DVT, with an incidence rate of 44.14%. Among the 655 patients with DVT, 558 had distal DVT, 97 had proximal DVT, with incidence rates of 85.19% and 14.81%, respectively. In 360 (54.96%) of the patients, DVT occurred in the fractured extremity, 222(33.89%) in the bilateral and 73(11.15%) in the non-fractured extremity. Patients diagnosed with DVT had no complained clinical symptom, and none of them developed PE. Participants in the highest quartile of serum albumin level (Q4) were younger than those in the lower quartiles (Q1–Q3) with a statistically significant difference (*P* < 0.001). The sex distribution also varied significantly, with a lower proportion of men in Q4 than in Q1 (*P* = 0.006). However, the time from injury to hospitalization was significantly shorter in Q4 than in Q1 (*P* < 0.001). The prevalence of diabetes was significantly higher in Q4 than in Q1(*P* < 0.001). Smoking also differed among groups, with the highest proportion of smokers in Q1 (*P* = 0.039). The baseline characteristics of the patients are summarized in Table 1.

**Table 1:** Baseline characteristics of participants.

Variables	Total (n = 1484)	Q1 (n = 370)	Q2 (n = 371)	Q3 (n = 372)	Q4 (n = 371)	*P*
Age, (years)	85.7 ± 4.2	86.1 ± 4.2	86.3 ± 4.5	85.5 ± 4.0	85.0 ± 3.9	< 0.001
Sex, n (%)						0.006
Male	366 (24.7)	110 (29.7)	92 (24.8)	95 (25.5)	69 (18.6)	
Female	1118 (75.3)	260 (70.3)	279 (75.2)	277 (74.5)	302 (81.4)	
Marital status, n (%)						0.396
Married	820 (55.3)	196 (53)	197 (53.1)	213 (57.3)	214 (57.7)	
Unmarried	3 (0.2)	2 (0.5)	0 (0)	1 (0.3)	0 (0)	
Widowed	661 (44.5)	172 (46.5)	174 (46.9)	158 (42.5)	157 (42.3)	
Time from injury to hospital, (hours)	12.0 (6.0, 48.0)	24.0 (8.2, 96.0)	24.0 (8.0, 72.0)	10.0 (5.0, 24.0)	7.0 (4.0, 24.0)	< 0.001
Hypertension, n (%)						0.054
No	701 (47.2)	183 (49.5)	187 (50.4)	178 (47.8)	153 (41.2)	
Yes	783 (52.8)	187 (50.5)	184 (49.6)	194 (52.2)	218 (58.8)	
Diabetes, n (%)						< 0.001
No	1160 (78.2)	309 (83.5)	301 (81.1)	284 (76.3)	266 (71.7)	
Yes	324 (21.8)	61 (16.5)	70 (18.9)	88 (23.7)	105 (28.3)	
Coronary artery disease, n (%)						0.204
No	1017 (68.5)	250 (67.6)	265 (71.4)	241 (64.8)	261 (70.4)	
Yes	467 (31.5)	120 (32.4)	106 (28.6)	131 (35.2)	110 (29.6)	
Renal insufficiency, n (%)						0.929
No	1355 (91.3)	335 (90.5)	341 (91.9)	340 (91.4)	339 (91.4)	
Yes	129 (8.7)	35 (9.5)	30 (8.1)	32 (8.6)	32 (8.6)	
History of VTE, n (%)						0.5
No	1469 (99.0)	366 (98.9)	368 (99.2)	370 (99.5)	365 (98.4)	
Yes	15 (1.0)	4 (1.1)	3 (0.8)	2 (0.5)	6 (1.6)	
Smoking, n (%)						0.039
No	1433 (96.6)	350 (94.6)	363 (97.8)	357 (96)	363 (97.8)	
Yes	51 (3.4)	20 (5.4)	8 (2.2)	15 (4)	8 (2.2)	
Alcoholism, n (%)						0.299
No	1461 (98.5)	362 (97.8)	365 (98.4)	365 (98.1)	369 (99.5)	
Yes	23 (1.5)	8 (2.2)	6 (1.6)	7 (1.9)	2 (0.5)	
PLT count, ×10^9^/L	196.8 ± 82.9	201.1 ± 92.7	196.9 ± 86.1	195.0 ± 78.8	194.1 ± 72.7	0.67
FIB, (g/L)	3.7 ± 0.9	3.8 ± 1.0	3.8 ± 1.0	3.7 ± 0.8	3.5 ± 0.8	< 0.001
TT, (s)	15.1 ± 3.9	15.2 ± 5.0	15.1 ± 1.7	15.2 ± 5.5	14.9 ± 1.6	0.566
APTT, (s)	31.4 ± 5.7	31.6 ± 6.6	31.8 ± 5.6	31.6 ± 5.7	30.8 ± 4.6	0.063
PT, (s)	12.5 ± 1.4	12.5 ± 1.3	12.7 ± 1.5	12.6 ± 1.7	12.4 ± 1.2	0.014
D-dimer, (μg/ml)	2.9 (1.7, 6.0)	2.7 (1.6, 5.4)	2.6 (1.5, 5.4)	2.8 (1.6, 5.8)	3.9 (2.0, 7.3)	< 0.001
TC, (mg/dL)	3.9 ± 1.0	3.4 ± 1.0	3.8 ± 0.8	4.0 ± 0.9	4.4 ± 1.0	< 0.001
TG, (mg/dL)	1.0 ± 0.5	0.9 ± 0.4	1.0 ± 0.4	1.1 ± 0.5	1.2 ± 0.7	< 0.001
DVT, n (%)						0.002
No	829 (55.9)	188 (50.8)	193 (52)	213 (57.3)	235 (63.3)	
Yes	655 (44.1)	182 (49.2)	178 (48)	159 (42.7)	136 (36.7)	

Abbreviations: PLT, Platelet Count; FIB, Fibrinogen; TT, thrombin time; APTT, activated partial thromboplastin time; PT, prothrombin time; TC, total cholesterol; TG, triglyceride

### Association between serum albumin and DVT

Table 2 presents the results of the multivariable logistic regression analyses. A significant inverse association was consistently observed between serum albumin levels and preoperative DVT across all models. As a continuous variable, in the fully adjusted Model 4, each 1 g/L higher serum albumin level was associated with 5% lower odds of preoperative DVT (OR, 0.95; 95%CI: 0.92–0.97, *P* < 0.001). When serum albumin was categorized by clinical threshold into two groups, using the group 1 as the reference group, albumin in the group 2 was showed a significant negative association in Model 4 (OR, 0.68; 95 CI%: 0.55–0.86, *P* = 0.001). When categorizing into serum albumin quartile, a significant association was still identified between albumin and preoperative DVT; the preoperative DVT in Q4 was significantly lower than that in Q1 (OR, 0.56; 95%CI: 0.40–0.78, *P* < 0.001) in the fully adjusted Model 4. The values of *P* for trend in all four models were both < 0.05 (Table 2). E-values were calculated to evaluate the potential influence of unmeasured confounding factors on the association between serum albumin levels and preoperative DVT using two group-based multivariable regression models. The E-value was 1.72.

**Table 2:** Multivariate logistic regression of the association between albumin and preoperative DVT.

Variable	Model 1		Model 2		Model 3		Model 4	
	OR (95%CI)	*P-*value	OR (95%CI)	*P-*value	OR(95%CI)	*P-*value	OR (95%CI)	*P-*value
Albumin per 1g/L	0.95 (0.93 ~ 0.98)	<0.001	0.95 (0.92 ~ 0.97)	<0.001	0.95 (0.92 ~ 0.97)	<0.001	0.95 (0.92 ~ 0.97)	<0.001
Group 1 (18.1-34.9)	1(Ref)		1(Ref)		1(Ref)		1(Ref)	
Group 2 (35-63.6)	0.7 (0.57 ~ 0.87)	0.001	0.68 (0.55 ~ 0.84)	<0.001	0.68 (0.55 ~ 0.85)	0.001	0.68 (0.55 ~ 0.86)	0.001
Quartile								
Q1 (18.1-32.80)	1(Ref)		1(Ref)		1(Ref)		1(Ref)	
Q2 (32.81-35.67)	0.95 (0.71 ~ 1.27)	0.742	0.95 (0.71 ~ 1.27)	0.75	0.98 (0.73 ~ 1.31)	0.882	0.96 (0.72 ~ 1.3)	0.802
Q3 (35.68-38.13)	0.77 (0.58 ~ 1.03)	0.078	0.75 (0.56 ~ 1.01)	0.055	0.76 (0.56 ~ 1.02)	0.067	0.75 (0.55 ~ 1.01)	0.061
Q4 (38.14-63.6)	0.6 (0.45 ~ 0.8)	0.001	0.57 (0.42 ~ 0.77)	<0.001	0.57 (0.42 ~ 0.78)	<0.001	0.56 (0.40 ~ 0.78)	<0.001
*P* for Trend		<0.001		<0.001		<0.001		<0.001

Model 1: unadjusted.

Model 2: adjust for age, sex, marital.

Model 3: age, sex, marital, hypertension, diabetes, coronary artery disease, renal insufficiency, history of VTE, smoking, alcoholism, time from injury to hospital.

Model 4: age, sex, marital, hypertension, diabetes, coronary artery disease, renal insufficiency, history of VTE, smoking, alcoholism, time from injury to hospital, TC, TG.

**Fig 2:**
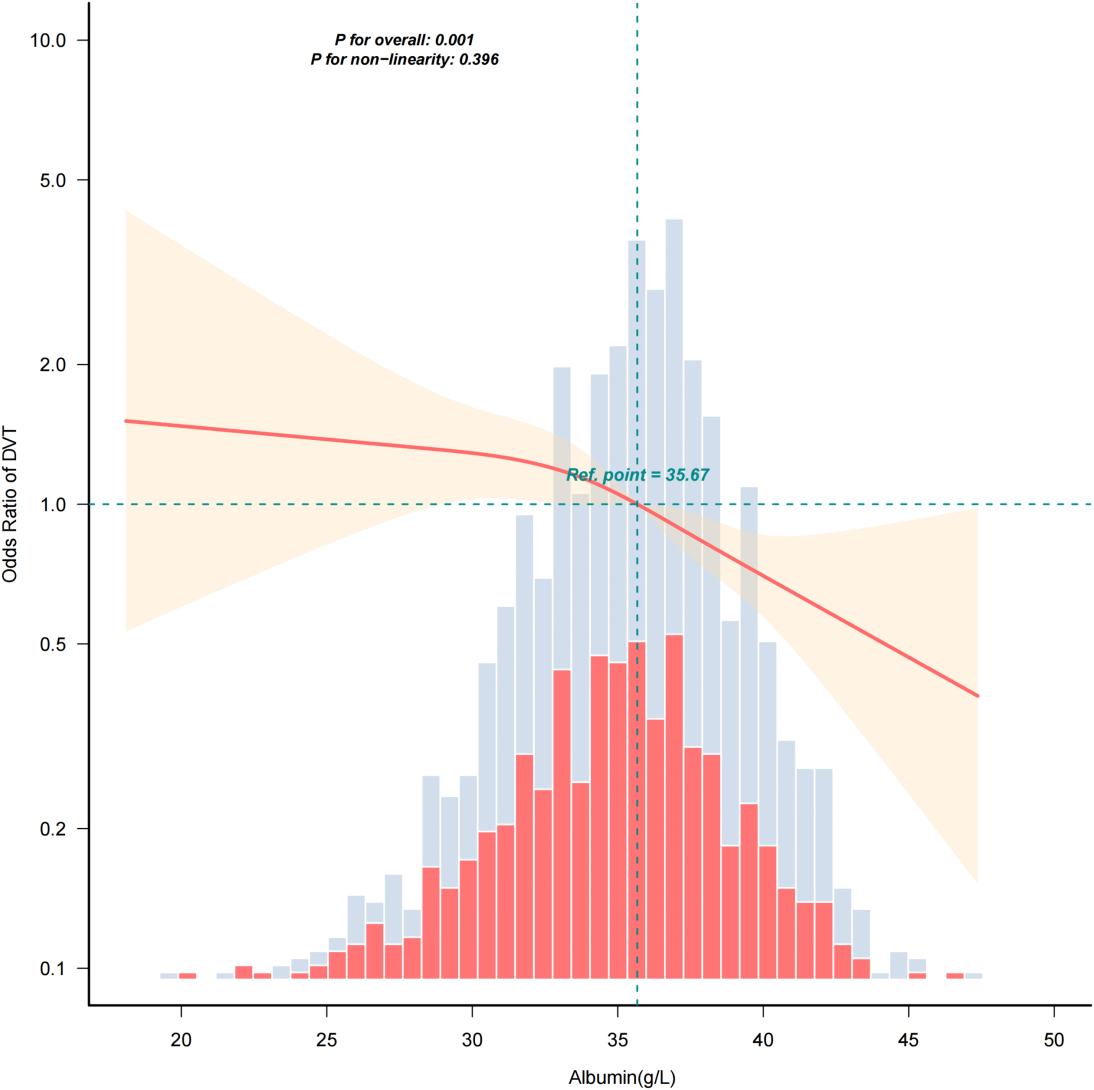
Dose-response relationship between albumin and DVT. Adjusted for age, sex, marital, time from injury to hospital, hypertension, diabetes, coronary artery disease, renal insufficiency, history of VTE, smoking, alcoholism, TC, TG. Only 99.9% data was used.

The RCS regression analysis revealed a negative linear dose – response relationship between serum albumin and preoperative DVT. A negative linear association between albumin concentration and DVT prevalence was identified; the overall association was statistically significant (*P* < 0.001), and the test for nonlinearity was not significant (*P* = 0.272) (Fig 2).

### Subgroup analyses

**Fig 3:**
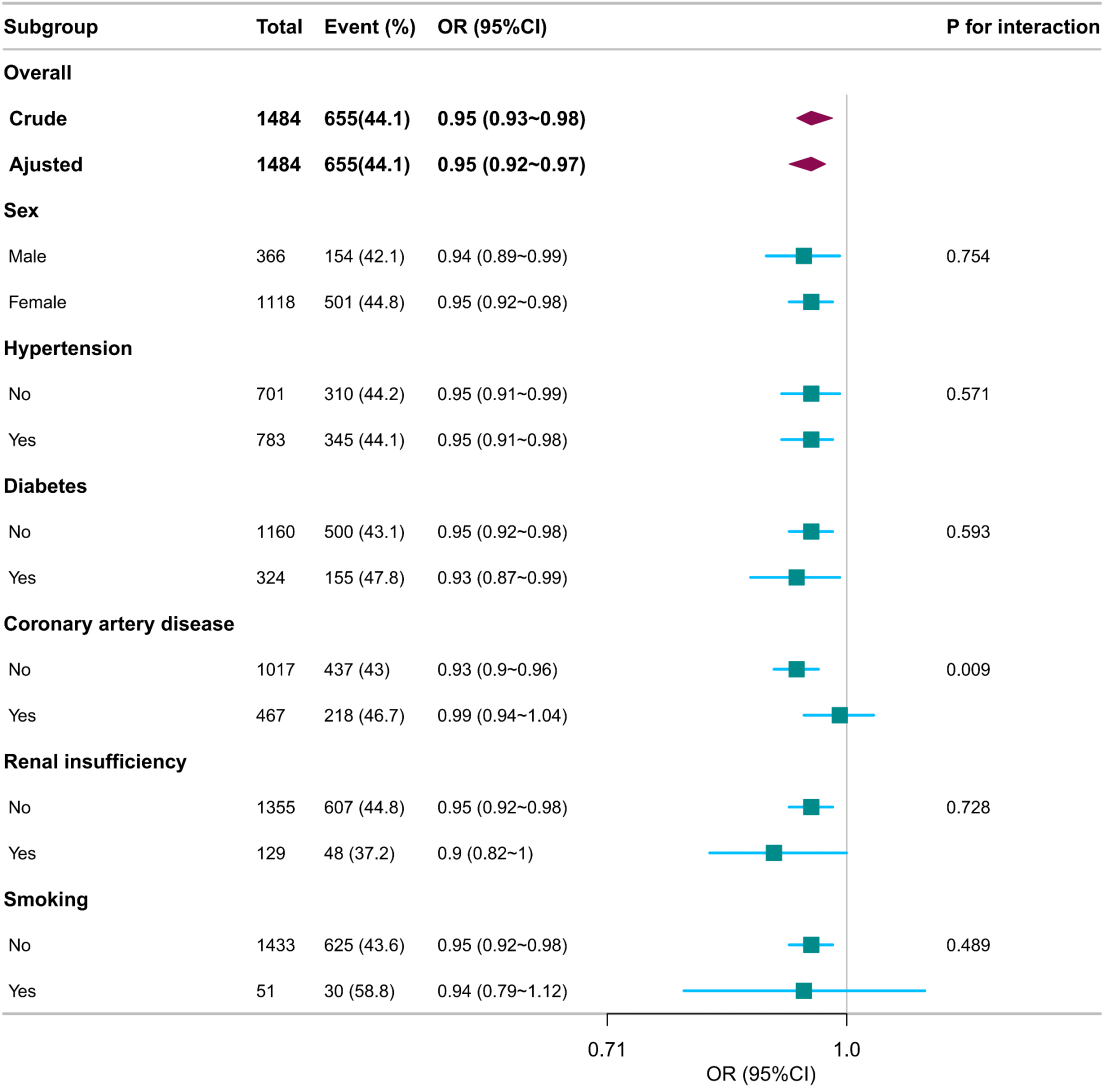
Forest plot. Subgroup and stratified analyses of the association between serum albumin and preoperative DVT. Adjusted for age, sex, marital, time from injury to hospital, hypertension, diabetes, coronary artery disease, renal insufficiency, history of VTE, smoking, alcoholism, TC, TG.

We conducted stratified and interaction analyses to evaluate the consistency of the association between serum albumin and preoperative DVT in various subgroup, including sex, hypertension, diabetes, coronary artery disease, renal insufficiency and smoking. The subgroup analysis revealed consistent inverse associations between serum albumin and preoperative DVT across various demographic and clinical subgroups. Across all subgroups, the interaction *P* values indicated no significant effect modification, except coronary heart disease (*P* for interaction = 0.009), suggesting that the inverse relationship between serum albumin and preoperative DVT was consistent (Fig 3).

### Sensitivity analysis

**Fig 4:**
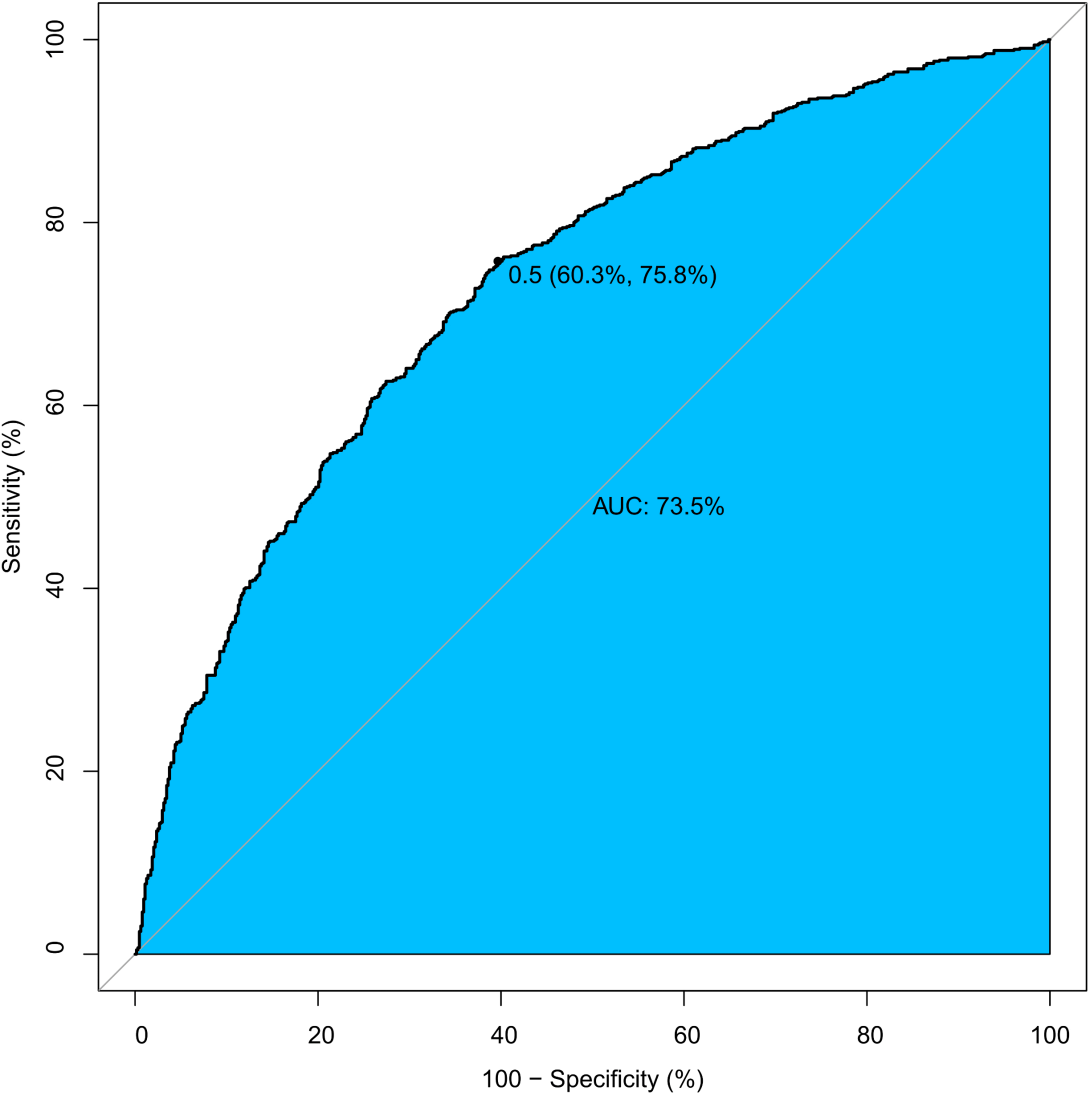
Propensity score matching ROC curve.

Furthermore, to reduce confounding by covariates and test the robustness of our results, several sensitivity analyses were also conducted. First, propensity score matching (PSM) employing the nearest-neighbor algorithm was implemented. Covariates were matched at a 1:1 ratio across groups, with the caliper width set at 0.20 SD. Standardized mean differences were then calculated to assess between-group characteristic differences, and 946 patients successfully matched. The multivariate logistic regression analysis revealed robust results under the PSM and PSM-adjusted models (Table 3; Fig 4 and S1 Fig). Second, we performed an additional analysis (Model 5) that further adjusted for PLT, FIB, TT, APTT, PT, and D-dimer. The results of the multivariate Logistic analysis were confirmed to be stable (S1 Table). Third, to evaluate the consistency of the association across DVT subtypes, we separately analyzed the association of albumin with distal DVT and proximal DVT. The results were consistent with the main findings, supporting the robustness of our conclusions (S2 and S3 Tables). Fourth, a sensitivity analysis was conducted by excluding patients with documented recent use of anticoagulant or antiplatelet medicines to evaluate the robustness of the primary findings. As shown in S4 Table, the association between serum albumin between preoperative DVT remained statistically significant and virtually unchanged compared with the primary analysis.

**Table 3:** Propensity score matching.

Models	OR (95%CI)	P value
Unmatched. crude	0.7 (0.57 ~ 0.87)	0.001
Multivariable. adjusted	0.68 (0.55 ~ 0.86)	0.001
Propensity Score. adjusted	0.7 (0.56 ~ 0.87)	0.002
Propensity Score. Matched	0.96 (0.93 ~ 0.996)	0.028
Weighted.PA	0.7 (0.54 ~ 0.89)	0.005
Weighted. Ow	0.7 (0.51 ~ 0.96)	0.026

## Discussion

A linear relationship between serum albumin concentration and preoperative DVT occurrence was identified. Elevated albumin levels correlated with a reduced incidence of preoperative DVT. Specifically, after adjusting for confounders, each 1 g/L higher serum albumin level was associated with 5% lower odds of preoperative DVT. When compared to the lowest quartile (Q1), patients in the Q4 albumin group exhibited significantly lower DVT risk, with a reduction of 44%. Clinically, serum albumin can be used as a useful preoperative marker to identify patients with a higher risk of DVT. Both the sensitivity and stratified analyses confirmed that the association between albumin and preoperative DVT remained robust. In the stratified analyses, significant interaction effects were observed for coronary heart disease but not for other variables. Stratified analysis showed a significant interaction effect in coronary heart disease, whereas no such phenomenon was observed in other variables. Thus, the association between albumin level and preoperative DVT may vary among patients with coronary heart disease. The potential modifying effect of heart disease on this association warrants further investigation.

Our findings showed preoperative DVT prevalence is 44.14%, including 85.19% of patients with distal DVT. Zuo [31] reported that 20.1% of patients older than 60 years had DVT on admission after intertrochanteric fracture, whereas Fei et al. [32] found a preoperative DVT rate of 37.6% in their retrospective study of 218 patients aged 16 years or older with intertrochanteric fractures. This might be because all patients included in this study were aged 80 years or older; advanced age is closely associated with a higher incidence of preoperative DVT, a risk further compounded by the poor nutritional status common in elderly patients and by the longer time from injury to hospitalization.

Meanwhile, several studies have demonstrated an association between serum albumin and DVT after hip fracture. By multivariate analysis, Zuo confirmed that a decreased serum albumin level < 31.7 g/L was an independent risk factor associated with DVT in bilateral lower extremities after intertrochanteric fracture in patients aged of ≥ 60 years [33]. Zhao has also showed that a serum albumin level < 35 g/L was significantly associated with the development of preoperative DVT in patients aged > 65 years with intertrochanteric fractures [22]. A meta-analysis has indicated that patients with serum albumin levels < 35 g/L were at an increased risk of DVT after hip fractures [34]. Huang has reported that serum albumin concentration is associated with mortality in geriatric patients with hip fractures and observed a linear association between serum albumin concentration and mortality in geriatric patients with hip fractures [35]. YiLun Wu has also reported that serum albumin level was associated with preoperative DVT, which was consistent with the results of this study, except that the age was ≥ 65 years [19]. Tang et al. have reported that lower admission serum albumin levels were independently associated with higher 30-day readmission rates in older patients with hip fractures [36].

Hypoalbuminemia, defined as serum albumin levels < 35 g/L, is a common clinical disorder characterized by subnormal serum albumin levels [37]. Serum albumin is a protein synthesized by the liver, and because of diminished liver function, albumin production may decrease with age [38,39]. Albumin may increase blood volume, preserve plasma colloid osmotic pressure, and create soluble compounds for detoxification and transportation by combining with numerous insoluble tiny molecules in the body. It potentially indicates underlying issues such as inflammation, protein loss, or malnutrition, which are clinically linked to diverse adverse outcomes [40,41]. Our study demonstrates that admission hypoalbuminaemia is associated with preoperative DVT and may correlate with poor nutritional status and diminished physiological reserve. However, this association is unlikely to be causal. Rather, hypoalbuminaemia likely reflects the presence of malnutrition and systemic inflammation, which can impair wound healing, muscle strength, and immunity [42,43], and serves as an indicator of poor general health or a marker of a true cause of VTE, such as a hyperinflammatory or hypercoagulable state [44]. A study has reported that hypoalbuminemia associates with increased fibrinogen concentrations and platelet aggregation [45]. Since albumin is only produced in the liver, a low serum albumin level indicates poor liver reserve and may lack anticoagulant factors, thereby increasing the risk of DVT [46]. Clinical studies in patients with liver cirrhosis have shown that hypoalbuminemia is closely related to an increased occurrence of DVT [47]. Similarly, hypoalbuminemia can be caused by loss of renal function. Studies have shown that low serum albumin can significantly predict the occurrence of DVT in nephrotic syndrome [48]. Therefore, low albumin levels can serve as markers of a hypercoagulable state [44].

Preoperative treatment for hypoalbuminemia and malnutrition has shown promise in reducing readmission and postoperative complications, and the detection of hypoalbuminemia and timely medical intervention may reduce complication rates and enhance prognosis [49,50]. In a randomized study, individuals with hip fractures who received specialized preoperative nutritional care had higher serum albumin levels and fewer sequelae [51]. Another trial used an integrative nutrition program that began at hospital admission, which greatly increased albumin levels and decreased complication rate of complications [52]. According to these findings, prompt dietary optimization and albumin repletion perioperatively may help attenuate readmission risk. Additional studies are necessary to identify the most effective approaches and optimal timeframe for nutritional support prior to surgery. Some ramifications for clinical practice are as follows. First and foremost, standardizing albumin monitoring is essential for the regular evaluation of older patients with hip fractures, especially those aged ≥80 years. Second, to predict long-term prognosis, albumin levels must be incorporated into preoperative risk assessment instruments.

This study has some limitations. First, the observational nature of this study precludes the establishment of a causal relationship. Second, potential confounding factors might not have been fully accounted for; despite model adjustments, we attempted to control for confounding factors and further estimated the impact of unmeasured confounding factors on the results using the E-value. Third, this study investigated the clinical implications of dynamic fluctuations in serum albumin levels and identified the optimal time frame for intervention, particularly concerning the rapid depletion observed following hip fracture events. Finally, this research employed a single-center design, and the external validity of these findings requires further validation.

## Conclusions

A significant inverse association was identified between serum albumin and preoperative DVT in patients aged ≥80 years with intertrochanteric fractures. The result has important implications for the development of clinical guidelines and updates in the management of patients with hip fractures. However, further validation and confirmation of our findings are warranted to strengthen the evidence base in this area.

## Supporting information

S1 Fig The Love plot of the Propensity Score Matched. (TIF)

S1 Table Multivariate logistic regression of the association between albumin and preoperative DVT. Model 1: unadjusted. Model 2: adjust for age, sex, marital. Model 3: age, sex, marital, hypertension, diabetes, coronary artery disease, renal insufficiency, history of VTE, smoking, alcoholism, time from injury to hospital. Model 4:age, sex, marital, hypertension, diabetes, coronary artery disease, renal insufficiency, history of VTE, smoking, alcoholism, time from injury to hospital, TC, TG.
(DOCX)

S2 Table Multivariate logistic regression of the association between albumin and preoperative distal DVT. Model 1: unadjusted. Model 2: adjust for age, sex, marital. Model 3: age, sex, marital, hypertension, diabetes, coronary artery disease, renal insufficiency, history of VTE, smoking, alcoholism, time from injury to hospital. Model 4: age, sex, marital, hypertension, diabetes, coronary artery disease, renal insufficiency, history of VTE, smoking, alcoholism, time from injury to hospital, TC, TG.
(DOCX)

S3 Table Multivariate logistic regression of the association between albumin and preoperative proximal DVT. Model 1: unadjusted. Model 2: adjust for age, sex, marital. Model 3: age, sex, marital, hypertension, diabetes, coronary artery disease, renal insufficiency, history of VTE, smoking, alcoholism, time from injury to hospital. Model 4: age, sex, marital, hypertension, diabetes, coronary artery disease, renal insufficiency, history of VTE, smoking, alcoholism, time from injury to hospital, TC, TG.
(DOCX)

S4 Table Multivariate logistic regression of the association between albumin and preoperative DVT. Model 1: unadjusted. Model 2: adjust for age, sex, marital. Model 3: age, sex, marital, hypertension, diabetes, coronary artery disease, renal insufficiency, history of VTE, smoking, alcoholism, time from injury to hospital. Model 4: age, sex, marital, hypertension, diabetes, coronary artery disease, renal insufficiency, history of VTE, smoking, alcoholism, time from injury to hospital, TC, TG.
(DOCX)
