# Supplementary material for: Association between serum albumin and preoperative Deep Vein Thrombosis in patients aged 80 and over with intertrochanteric fracture: A retrospective observational study
Source: PLoS One. 2026 Aug 14;21(8):e0345895. doi: 10.1371/journal.pone.0345895 (PMC13475855; doi:10.1371/journal.pone.0345895)
Supplement: Supplementary file 1 [file pone.0345895.s002.docx]

**S1 Table Multivariate logistic regression of the association between albumin and preoperative DVT**

| **Variable** | **Model 1** | | **Model 2** | | **Model 3** | | **Model 4** | | **Model 5** | |
| --- | --- | --- | --- | --- | --- | --- | --- | --- | --- | --- |
|  | **OR (95%CI)** | ***P*-value** | **OR (95%CI)** | ***P*-value** | **OR**  **(95%CI)** | ***P*-value** | **OR (95%CI)** | ***P*-value** | **OR (95%CI)** | ***P*-value** |
| Albumin  per 1g/L | 0.95 (0.93~0.98) | <0.001 | 0.95 (0.92~0.97) | <0.001 | 0.95 (0.92~0.97) | <0.001 | 0.95 (0.92~0.97) | <0.001 | 0.94 (0.91~0.97) | <0.001 |
| Group 1  (18.1-34.9) | 1(Ref) |  | 1(Ref) |  | 1(Ref) |  | 1(Ref) |  | 1(Ref) |  |
| Group 2  (35-63.6) | 0.7 (0.57~0.87) | 0.001 | 0.68 (0.55~0.84) | <0.001 | 0.68 (0.55~0.85) | 0.001 | 0.66 (0.53~0.83) | <0.001 | 0.66 (0.53~0.83) | <0.001 |
| Quartile |  |  |  |  |  |  |  |  |  |  |
| Q1  (18.1-32.80) | 1(Ref) |  | 1(Ref) |  | 1(Ref) |  | 1(Ref) |  | 1(Ref) |  |
| Q2  (32.81-35.67) | 0.95 (0.71~1.27) | 0.742 | 0.95 (0.71~1.27) | 0.75 | 0.98 (0.73~1.31) | 0.882 | 0.96 (0.72~1.3) | 0.802 | 0.94 (0.7~1.27) | 0.684 |
| Q3  (35.68-38.13) | 0.77 (0.58~1.03) | 0.078 | 0.75 (0.56~1.01) | 0.055 | 0.76 (0.56~1.02) | 0.067 | 0.75 (0.55~1.01) | 0.061 | 0.72 (0.53~0.97) | 0.033 |
| Q4  (38.14-63.6) | 0.6 (0.45~0.8) | 0.001 | 0.57 (0.42~0.77) | <0.001 | 0.57 (0.42~0.78) | <0.001 | 0.56 (0.4~0.78) | <0.001 | 0.52 (0.37~0.73) | <0.001 |
| *P* for Trend |  | <0.001 |  | <0.001 |  | <0.001 |  | <0.001 |  | <0.001 |

Model 1: unadjusted.

Model 2: adjust for age,sex,marital.

Model 3:age, sex,marital, hypertension, diabetes, coronary artery disease, renal insufficiency, history of VTE, smoking, alcoholism, time from injury to hospital.

Model 4:age, sex, marital, hypertension, diabetes, coronary artery disease, renal insufficiency, history of VTE, smoking, alcoholism, time from injury to hospital, TC, TG.
