# Supplementary material for: Association between serum albumin and preoperative Deep Vein Thrombosis in patients aged 80 and over with intertrochanteric fracture: A retrospective observational study
Source: PLoS One. 2026 Aug 14;21(8):e0345895. doi: 10.1371/journal.pone.0345895 (PMC13475855; doi:10.1371/journal.pone.0345895)
Supplement: Supplementary file 2 [file pone.0345895.s003.docx]

**S2 Table Multivariate logistic regression of the association between albumin and preoperative distal DVT**

| **Variable** | **Model 1** | | **Model 2** | | **Model 3** | | **Model 4** | |
| --- | --- | --- | --- | --- | --- | --- | --- | --- |
|  | **OR**  **(95%CI)** | ***P-*value** | **OR (95%CI)** | ***P-*value** | **OR (95%CI)** | ***P-*value** | **OR**  **(95%CI)** | ***P-*value** |
| Albumin  per 1g/L | 0.96 (0.94~0.99) | 0.002 | 0.96 (0.93~0.98) | 0.001 | 0.95 (0.93~0.98) | 0.001 | 0.95 (0.93~0.98) | 0.002 |
| Group 1  (18.1-34.9) | 1(Ref) |  | 1(Ref) |  | 1(Ref) |  | 1(Ref) |  |
| Group 2  (35-63.6) | 0.76 (0.61~0.95) | 0.014 | 0.73 (0.59~0.92) | 0.006 | 0.73 (0.58~0.91) | 0.006 | 0.73 (0.58~0.93) | 0.009 |
| Quartile |  |  |  |  |  |  |  |  |
| Q1  (18.1-32.90) | 1(Ref) |  | 1(Ref) |  | 1(Ref) |  | 1(Ref) |  |
| Q2  (32.91-35.80) | 0.95 (0.7~1.29) | 0.764 | 0.96 (0.71~1.3) | 0.784 | 0.97 (0.72~1.32) | 0.85 | 0.95 (0.7~1.3) | 0.755 |
| Q3  (35.81-38.20) | 0.83 (0.61~1.12) | 0.226 | 0.76 (0.56~1.03) | 0.08 | 0.76 (0.56~1.03) | 0.08 | 0.8 (0.58~1.1) | 0.164 |
| Q4  (38.21-63.6) | 0.66 (0.49~0.9) | 0.008 | 0.63 (0.46~0.86) | 0.003 | 0.63 (0.45~0.86) | 0.004 | 0.62 (0.44~0.87) | 0.006 |
| *P* for Trend |  | 0.005 |  | 0.001 |  | 0.001 |  | 0.003 |

Model 1: unadjusted.

Model 2: adjust for age, sex, marital.

Model 3:age, sex,marital, hypertension, diabetes, coronary artery disease, renal insufficiency, history of VTE, smoking, alcoholism, time from injury to hospital.

Model 4:age, sex, marital, hypertension, diabetes, coronary artery disease, renal insufficiency, history of VTE, smoking, alcoholism, time from injury to hospital, TC, TG.
