# Supplementary material for: Association between serum albumin and preoperative Deep Vein Thrombosis in patients aged 80 and over with intertrochanteric fracture: A retrospective observational study
Source: PLoS One. 2026 Aug 14;21(8):e0345895. doi: 10.1371/journal.pone.0345895 (PMC13475855; doi:10.1371/journal.pone.0345895)
Supplement: Supplementary file 3 [file pone.0345895.s004.docx]

**S3 Table Multivariate logistic regression of the association between albumin and preoperative proximal DVT**

| **Variable** | **Model 1** | | **Model 2** | | **Model 3** | | **Model 4** | |
| --- | --- | --- | --- | --- | --- | --- | --- | --- |
|  | **OR (95%CI)** | ***P-*value** | **OR (95%CI)** | ***P-*value** | **OR (95%CI)** | ***P-*value** | **OR (95%CI)** | ***P-*value** |
| Albumin  per 1g/L | 0.95 (0.93~0.98) | <0.001 | 0.95 (0.93~0.98) | <0.001 | 0.95 (0.93~0.98) | 0.001 | 0.95 (0.92~0.98) | 0.001 |
| Group 1  (18.1-34.9) | 1(Ref) |  | 1(Ref) |  | 1(Ref) |  | 1(Ref) |  |
| Group 2  (35-63.6) | 0.71 (0.57~0.88) | 0.001 | 0.69 (0.56~0.85) | 0.001 | 0.69 (0.55~0.87) | 0.001 | 0.69 (0.55~0.87) | 0.002 |
| Quartile |  |  |  |  |  |  |  |  |
| Q1  (18.1-32.80) | 1(Ref) |  | 1(Ref) |  | 1(Ref) |  | 1(Ref) |  |
| Q2  (32.81-35.62) | 0.98 (0.73~1.31) | 0.88 | 0.98 (0.73~1.32) | 0.9 | 1.01 (0.75~1.36) | 0.952 | 1 (0.74~1.36) | 0.995 |
| Q3  (35.63-38.13) | 0.78 (0.58~1.06) | 0.111 | 0.77 (0.57~1.03) | 0.081 | 0.78 (0.57~1.05) | 0.105 | 0.77 (0.56~1.05) | 0.104 |
| Q4  (38.14-63.6) | 0.61 (0.45~0.83) | 0.001 | 0.59 (0.43~0.8) | 0.001 | 0.6 (0.44~0.82) | 0.001 | 0.59 (0.42~0.82) | 0.002 |
| *P* for Trend |  | <0.001 |  | <0.001 |  | <0.001 |  | 0.001 |

Model 1: unadjusted.

Model 2: adjust for age, sex, marital.

Model 3:age, sex, marital, hypertension, diabetes, coronary artery disease, renal insufficiency, history of VTE, smoking, alcoholism,time from injury to hospital.

Model 4:age, sex, marital, hypertension, diabetes, coronary artery disease, renal insufficiency, history of VTE, smoking, alcoholism, time from injury to hospital, TC, TG.
