# Supplementary material for: Association between serum albumin and preoperative Deep Vein Thrombosis in patients aged 80 and over with intertrochanteric fracture: A retrospective observational study
Source: PLoS One. 2026 Aug 14;21(8):e0345895. doi: 10.1371/journal.pone.0345895 (PMC13475855; doi:10.1371/journal.pone.0345895)
Supplement: Supplementary file 4 [file pone.0345895.s005.docx]

**S4 Table Multivariate logistic regression of the association between albumin and preoperative DVT**

| **Variable** | **Model 1** | | **Model 2** | | **Model 3** | | **Model 4** | |
| --- | --- | --- | --- | --- | --- | --- | --- | --- |
|  | **OR (95%CI)** | ***P-*value** | **OR (95%CI)** | ***P-*value** | **OR (95%CI)** | ***P-*value** | **OR (95%CI)** | ***P-*value** |
| Albumin  per 1g/L | 0.95 (0.93~0.98) | <0.001 | 0.95 (0.93~0.98) | <0.001 | 0.95 (0.93~0.98) | 0.001 | 0.95 (0.92~0.98) | 0.001 |
| Group 1  (18.1-34.9) | 1(Ref) |  | 1(Ref) |  | 1(Ref) |  | 1(Ref) |  |
| Group 2  (35-63.60) | 0.71 (0.57~0.88) | 0.001 | 0.69 (0.56~0.85) | 0.001 | 0.69 (0.55~0.87) | 0.001 | 0.69 (0.55~0.87) | 0.002 |
| Quartile |  |  |  |  |  |  |  |  |
| Q1  (18.1-32.80) | 1(Ref) |  | 1(Ref) |  | 1(Ref) |  | 1(Ref) |  |
| Q2  (32.81-35.67) | 0.97 (0.72~1.3) | 0.828 | 0.97 (0.72~1.31) | 0.846 | 1 (0.74~1.35) | 0.998 | 0.99 (0.73~1.34) | 0.954 |
| Q3  (35.68-38.13) | 0.8 (0.59~1.07) | 0.134 | 0.78 (0.57~1.05) | 0.098 | 0.79 (0.58~1.07) | 0.125 | 0.78 (0.57~1.07) | 0.123 |
| Q4  (38.14-63.60) | 0.61 (0.45~0.82) | 0.001 | 0.58 (0.43~0.79) | 0.001 | 0.59 (0.43~0.81) | 0.001 | 0.58 (0.42~0.82) | 0.002 |
| *P* for Trend |  | <0.001 |  | <0.001 |  | <0.001 |  | 0.001 |

Model 1: unadjusted.

Model 2: adjust for age, sex, marital.

Model 3: age, sex, marital, hypertension, diabetes, coronary artery disease, renal insufficiency, history of VTE, smoking, alcoholism, time from injury to hospital.

Model 4:age, sex, marital, hypertension, diabetes, coronary artery disease, renal insufficiency, history of VTE, smoking, alcoholism, time from injury to hospital, TC, TG.
